# In Vitro Toxicological Screening of Stable and Senescing Cultures of *Aphanizomenon*, *Planktothrix*, and *Raphidiopsis*

**DOI:** 10.3390/toxins12060400

**Published:** 2020-06-17

**Authors:** Łukasz Wejnerowski, Halina Falfushynska, Oksana Horyn, Inna Osypenko, Mikołaj Kokociński, Jussi Meriluoto, Tomasz Jurczak, Barbara Poniedziałek, Filip Pniewski, Piotr Rzymski

**Affiliations:** 1 Department of Hydrobiology, Institute of Environmental Biology, Faculty of Biology, Adam Mickiewicz University in Poznań, Uniwersytetu Poznańskiego 6, 61-614 Poznań, Poland; kok@amu.edu.pl; 2 Department of Orthopedagogy and Physical Therapy, Ternopil V. Hnatiuk National Pedagogical University, 46027 Ternopil, Ukraine; horynoi@tnpu.edu.ua (O.H.); osypenko09inna@gmail.com (I.O.); 3 Biochemistry, Faculty of Science and Engineering, Åbo Akademi University, Tykistökatu 6A, 20520 Turku, Finland; jussi.meriluoto@abo.fi; 4 United Nations Educational, Scientific and Cultural Organization (UNESCO) Chair on Ecohydrology and Applied Ecology, Faculty of Biology and Environmental Protection, University of Łódź, Banacha 12/16, 90-237 Łódź, Poland; tomasz.jurczak@biol.uni.lodz.pl; 5 Department of Environmental Medicine, Poznan University of Medical Sciences, Rokietnicka 8, 60-806 Poznań, Poland; bpon@ump.edu.pl; 6 Institute of Oceanography, University of Gdańsk, Al. Piłsudskiego 46, 81-378 Gdynia, Poland; filip.pniewski@ug.edu.pl

**Keywords:** *Aphanizomenon*, *Planktothrix*, *Raphidiopsis*, cyanobacteria, cyanotoxins, toxicological screening, in vitro experimental model

## Abstract

Toxicity of cyanobacteria is the subject of ongoing research, and a number of toxic metabolites have been described, their biosynthesis pathways have been elucidated, and the mechanism of their action has been established. However, several knowledge gaps still exist, e.g., some strains produce hitherto unknown toxic compounds, while the exact dynamics of exerted toxicity during cyanobacterial growth still requires further exploration. Therefore, the present study investigated the toxicity of extracts of nine freshwater strains of *Aphanizomenon gracile*, an *Aphanizomenon* sp. strain isolated from the Baltic Sea, a freshwater strain of *Planktothrix agardhii*, and two strains of *Raphidiopsis raciborskii* obtained from 25- and 70-day-old cultures. An in vitro experimental model based on *Cyprinus carpio* hepatocytes (oxidative stress markers, DNA fragmentation, and serine/threonine protein activity) and brain homogenate (cholinesterase activity) was employed. The studied extracts demonstrated toxicity to fish cells, and in general, all examined extracts altered at least one or more of considered parameters, indicating that they possess, to some degree, toxic potency. Although the time from which the extracts were obtained had a significant importance for the response of fish cells, we observed strong variability between the different strains and species. In some strains, extracts that originated from 25-day-old cultures triggered more harmful effects on fish cells compared to those obtained from 70-day-old cultures, whereas in other strains, we observed the opposite effect or a lack of a significant change. Our study revealed that there was no clear or common pattern regarding the degree of cyanobacterial bloom toxicity at a given stage of development. This means that young cyanobacterial blooms that are just forming can pose an equally toxic threat to aquatic vertebrates and ecosystem functioning as those that are stable or old with a tendency to collapse. This might be largely due to a high variability of strains in the bloom.

## 1. Introduction

Cyanobacterial blooms have garnered increased scientific interest because of their potential threat to aquatic biota, ecosystem functioning, and adverse effects on human health. Although these blooms have occurred before the impacts of humans on the aquatic environment [1,2,3], their frequency, intensity, range, and time of persistence have increased over the last few decades [4,5]. Anthropogenic eutrophication and climate change are two of the most influential factors that promote cyanobacterial blooms [6,7,8]. These blooms most often occur in months with the highest mean air temperature, although winter blooms in eutrophic lakes have been reported with increasing frequency [9,10,11,12,13].

Common bloom-forming cyanobacteria species encompass several genera, including *Aphanizomenon*, *Dolichospermum*, *Microcystis*, *Nodularia*, *Planktothrix*, *Raphidiopsis*, and *Trichodesmium*. The nutritional value of cyanobacteria for consumers, such as zooplankton, is generally low because their cells are deficient in sterols and fatty acids [14]; although, at low biomass, they can be a valuable source of food for consumers [15,16]. Filamentous forms and strains forming colonies with mucilaginous sheaths are challenging for grazers, particularly for filter-feeding zooplankters such as *Daphnia* [17]. Dense cyanobacterial blooms cause a significant decrease in transparency and availability of light to other phytoplankton representatives and aquatic macrophytes. Importantly, some bloom-forming cyanobacteria can produce a wide array of metabolites with toxicity to aquatic biota and humans [18,19], and release odor compounds [20]. Under chronic or acute exposure scenarios, cyanotoxins can bioaccumulate in biota, induce a wide range of toxic effects [21], and lead to high mortality among aquatic animals [22] as well as human intoxication [23]. On the other hand, a number of studies have shown that some strains do not produce any known cyanotoxins; the causative factors remain to be identified [24,25]. 

The harmfulness of cyanobacterial blooms is modified by their stage, state, and the contribution of the toxic subpopulation. At the initial stage of development, blooms usually demonstrate low harmfulness mainly because of their low biomass and, in some circumstances, due to their partial control by grazers [15,26,27,28] compared to dense, stable, or senescing blooms. The population dynamics and toxicity of a bloom are generally influenced by environmental factors, including mainly meteorological (as temperature and light conditions), chemical (as nutrients concentrations), and biological parameters (competition, bacterial activity, grazing). The impact of these factors is manifested by the selection of toxic and nontoxic populations and regulation of gene expression responsible for cyanotoxins synthesis [29]. However, it is not fully known whether and how the toxicity of different cyanobacteria is modified during bloom formation and senescence. Previous studies have shown conflicting results regarding the effect of growth cycles on toxicity. For example, Yunes and collaborators [30] reported higher saxitoxin (Stx) concentrations in the exponential phase of *Raphidiopsis brookii*, while conversely, other researchers [31,32] demonstrated higher Stx concentrations during the stationary phase of *Aphanizomenon* sp. and *Raphidiopsis raciborskii*, respectively. Cells at the logarithmic and early stationary states do not experience nutrient limitation like during the death phase at which the pool of nutrients is often exhausted. Moreover, during the death phase, the entire range of metabolites can be released by cell lysis [33]. On the other hand, various metabolites can be actively produced and released by intact cells to acquire available nutrients and sustain growth. For example, it has been suggested that cylindrospermopsin is involved in phosphate acquisition by triggering the release of alkaline phosphatase in other phytoplankton species [34,35]. However, some toxins, such as microcystins, can be released during necrosis and stress-induced programmed cell death to mediate the survival of the remaining cell population under unfavorable conditions [36].

The production of cyanotoxins in cyanobacteria is the highest at favorable conditions for growth, reaching the maximum at the late logarithmic growth phase [37,38,39]. In addition, senescing blooms are often accompanied by increased activity of heterotrophic bacteria [40] that can metabolize a range of exudates of cyanobacteria, including cyanotoxins and toxic metabolites [41,42]. Therefore, one hypothesis is that stable cyanobacterial blooms at the late exponential or early stationary growth phase are more harmful than the senescing ones. On the other hand, aging dense blooms are exposed to numerous stressors, including nutrient starvation, changes in physicochemical parameters of water (especially pH), and strong self-shading, which may induce morphological and physiological alterations in cyanobacterial cells, e.g., formation of sheaths or mucilaginous envelopes by *Planktothrix* [43] or increased microcystin production by *Microcystis* in response to nutrient starvation [44]. Moreover, excessive decay of cyanobacteria-derived organic matter during a bloom collapse often induces the depletion of dissolved oxygen and consequently leads to anoxic conditions [45]. In addition, an increased rate of apoptotic-like death of cyanobacterial cells in collapsing blooms means that the entire range of intracellular metabolites is released into the environment during cell lysis, and they can be as toxic and lethal as cyanotoxins alone [36]. Following these reports, an alternative hypothesis is that the senescing cyanobacterial blooms, due to the release of a wide array of molecules, are more harmful than younger cyanobacterial blooms.

The present study compared the toxicity of extracts obtained from thirteen dense cultures of filamentous cyanobacteria comprising *Aphanizomenon gracile* Lemmermann, *Aphanizomenon* sp., *Planktothrix agardhii* (Gomont) Anagnostidis & Komárek, and *Raphidiopsis raciborskii* (Woloszynska) Aguilera, Berrendero Gómez, Kaštovský, Echenique, and Salerno (basionym *Cylindrospermopsis raciborskii* Wołoszyńska Seenaya and Subba Raju) at an early and late stage of their development (25 and 70 days of cultivation, respectively). A battery of in vitro tests employing *Cyprinus carpio* cells was applied, including (i) oxidative stress indices: total glutathione (GSH) level, the activity of glutathione-S-transferase (GST) and catalase (CAT), total antioxidant capacity (TAC), lipid peroxidation (LPO), and protein carbonyl (PC) level in hepatocytes, (ii) DNA fragmentation (DSBs) in hepatocytes, (iii) serine/threonine protein phosphatase activity (PSP) in hepatocytes, and (iv) cholinesterase activity (ChE) in the brain homogenate. The extracts concentrations were standardized for the chlorophyll-a content in cyanobacterial cultures to allow for direct comparison of the observed effects.

The study provides new insights into the understanding of the toxicity profile of cyanobacteria at different stages of development.

## 2. Results

Neither HPLC-DAD nor HPLC-MS/MS revealed the presence of CYN in 12 of the 13 examined strains (Table 1). MC-RR, MC-YR, MC-LR, as well as demethyl group of MC-RR and MC-LR were detected only in *P. agardhii* SAG 6.89 [46]. Based on the characteristic spectra of absorption, no other MCs were identified in *P. agardhii* SAG 6.89 or five other strains. The observed response of parameters (oxidative stress markers, DNA strand breaks, serine/threonine protein phosphatases activity, and cholinesterase activity) employed in the toxicity evaluation revealed a dependence on the stage of culture development at which the studied extracts were obtained, the cyanobacterial strain, as well as the interaction between the stage of culture development and the strains (Table 2).

### 2.1. Oxidative Stress

The potency of cyanobacterial extracts from the two stages of development (25- and 70-day-old cultures) to induce oxidative stress in hepatocytes isolated from the common carp was investigated based on six parameters: the level of GSH, the activity of GST and CAT, total antioxidant capacity measured as Trolox equivalents, as well as direct outcomes of the increased intracellular levels of reactive oxygen species: protein carbonylation and lipid peroxidation (measured as thiobarbituric acid reactive substances; TBARS). 

**Figure 1:**
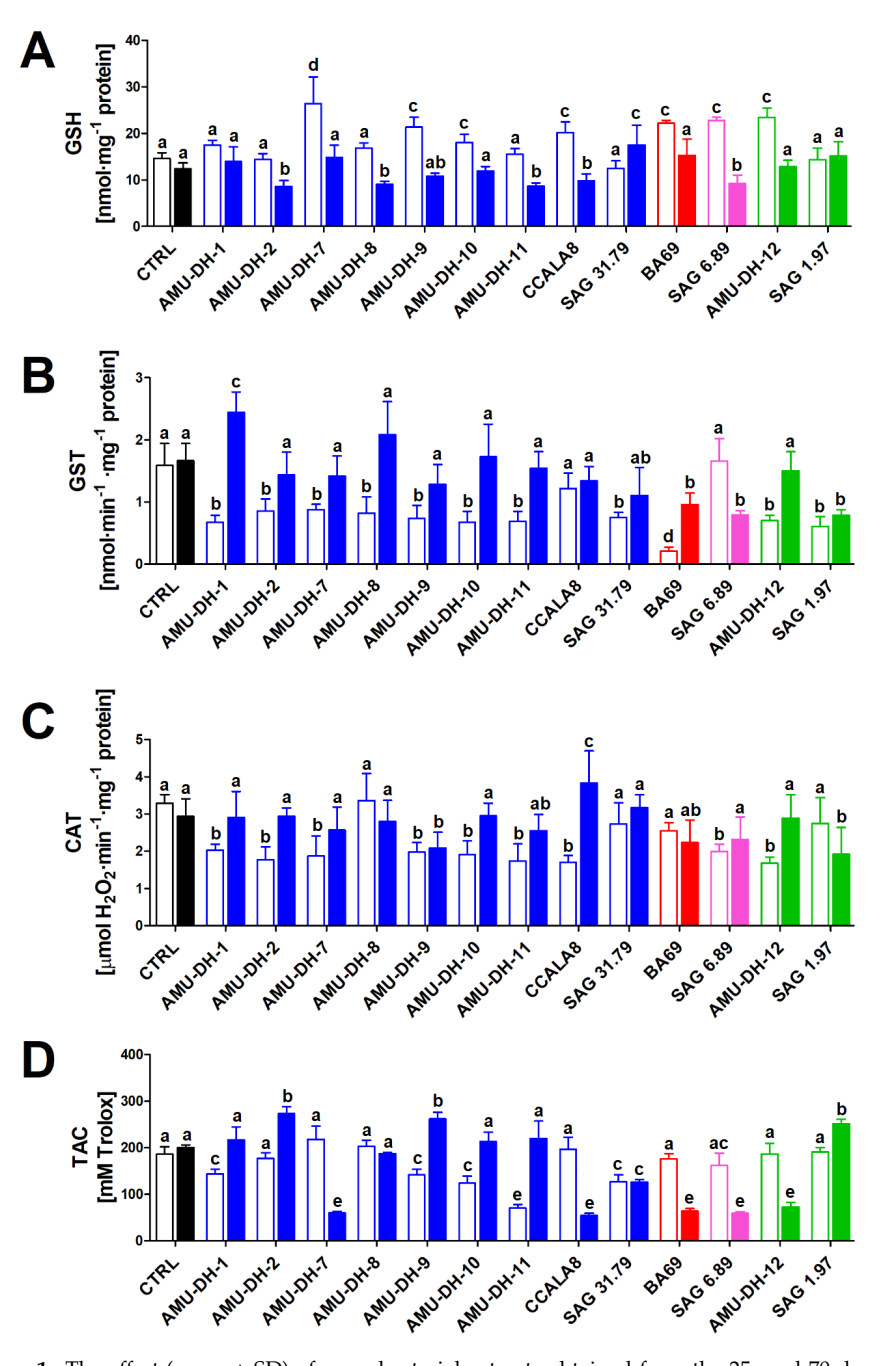
The effect (mean ± SD) of cyanobacterial extracts obtained from the 25- and 70-day-old cultures (open and filled bars, respectively) on glutathione level (GSH; **A**), glutathione S-transferase activity (GST; **B**), catalase activity (CAT; **C**), and total antioxidant capacity (TAC; **D**) in hepatocytes isolated from common carp (*n* = 8). CTRL—control (cells exposed to pure WC medium). Blue bars—*A. gracile* strains; red bars—*Aphanizomenon* sp. from the Baltic Sea; pink bars—*P. agardhii*; green bars—*R. raciborskii* strains. Different lowercase letters (a–e) above the bars indicate statistically significant difference between groups revealed by post hoc tests for pairwise comparisons (*p* < 0.05).

None of the studied cyanobacterial extracts obtained from the 25-day-old cultures caused a depletion of the total GSH content in carp hepatocytes, with an increase noted after treatment with extracts of selected strains of *A. gracile* (AMU-DH-7, AMU-DH-9, AMU-DH-10, CCALA8), *R. raciborskii* AMU-DH-12, *Aphanizomenon* sp. BA 69, and *P. agardhii* SAG 6.89. On the contrary, most *A. gracile* extracts from the 70-day-old cultures caused a depletion of the cellular GSH content—the only exceptions were *A. gracile* AMU-DH-1 and AMU-DH-7, for which no significant change compared to control was noted, and *A. gracile* SAG 31.79 for which an increase was observed. Extracts from the 70-day-old *R. raciborskii* strains and *Aphanizomenon* sp. BA 69 did not induce any changes in the GSH level (Figure 1).

Nearly all cyanobacterial extracts from the 25-day-old cultures (with the exception of *A. gracile* CCALA 8 and *P. agardhii* SAG 6.89) caused a significant decrease in the GST activity in carp hepatocytes. In the case of extracts from 70-day-old cultures, such an effect was only noted for *R. raciborskii* SAG 1.97, *Aphanizomenon* sp. BA 69 and *P. agardhii* SAG 6.89 (Figure 1).

Following the treatment of hepatocytes with extracts from the younger (25-day-old) cultures, a decrease in the CAT activity was noted for all of them with the exception of *A*. *gracile* AMU-DH-8 and SAG 31.79, and *Aphanizomenon* sp. BA69 for which no significant change was observed. An extract of only one strain, *A. gracile* CCALA8, from the older (70-day-old) cultures, induced an increase in the CAT activity. Most of the studied extracts from the older cultures did not cause any significant changes with the exception of *A. gracile* AMU-DH-9 and *R*. *raciborskii* SAG 1.97, for which a decrease in the enzyme activity was noted (Figure 1).

The TAC of hepatocytes displayed different responses to cyanobacterial extracts depending on the strain and stage of development. In extracts obtained from some of the *A. gracile* strains (AMU-DH-1, AMU-DH-9, AMU-DH-10, AMU-DH-11, and SAG 31.79), a decrease in the level of this parameter was observed. A similar effect was produced by the following extracts from the older cultures: *A. gracile* AMU-DH-7 and SAG 31.79, *Aphanizomenon* sp. BA 69, *P. agardhii* SAG 6.89, and *R. raciborskii* AMU-DH-12 (Figure 1).

**Figure 2:**
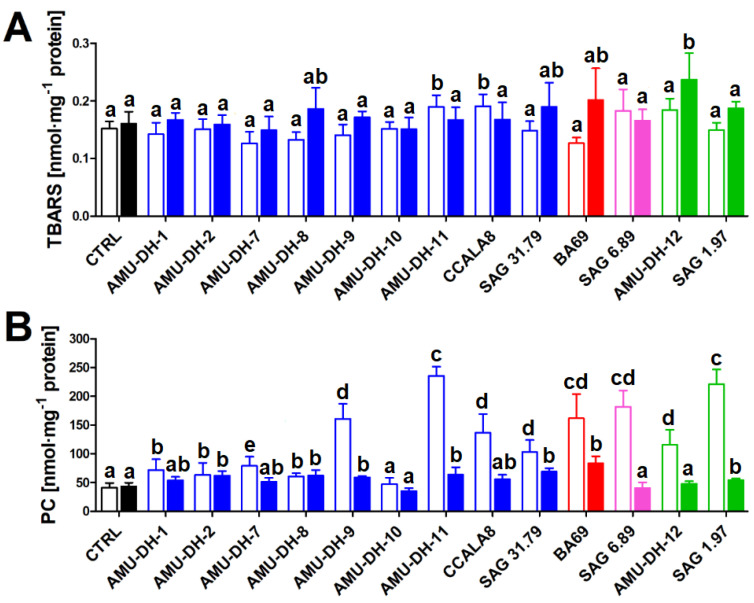
The effect (mean ± SD) of cyanobacterial extracts obtained from the 25- and 70-day-old cultures (open and filled bars, respectively) on lipid peroxidation (TBARS; **A**) and protein carbonylation (PC; **B**), in hepatocytes isolated from common carp (*n* = 8). CTRL—control (cells exposed to pure WC medium). Blue bars—*A. gracile* strains; red bars—*Aphanizomenon* sp. from the Baltic Sea; pink bars—*P. agardhii*; green bars—*R. raciborskii* strains. Different lowercase letters (a–d) above the bars indicate statistically significant difference between groups revealed by post hoc tests for pairwise comparisons (*p* < 0.05).

Most of the investigated extracts did not significantly affect lipid peroxidation (Figure 2). However, a relevant increase of TBARS was observed for the extracts obtained from the 25-day-old cultures of *A*. *gracile* AMU-DH-11 and CCALA 8, as well as from 70-day-old *R*. *raciborskii* AMU-DH-12. Exposure of carp hepatocytes to nearly all extracts resulted in an increase in the level of protein carbonyls; the most profound effects were noted for the treatments with extracts from the following 25-day-old cultures of *A. gracile* AMU-DH-9, AMU-DH-11 and CCALA 8, *Aphanizomenon* sp. BA 69, *P. agardhii* SAG 6.89, and *R. raciborskii* AMU-DH-12 and SAG 1.97. The only strain that did not induce any effect regardless of the stage of development was *A. gracile* AMU-DH-10 (Figure 2).

### 2.2. Genotoxicity

**Figure 3:**
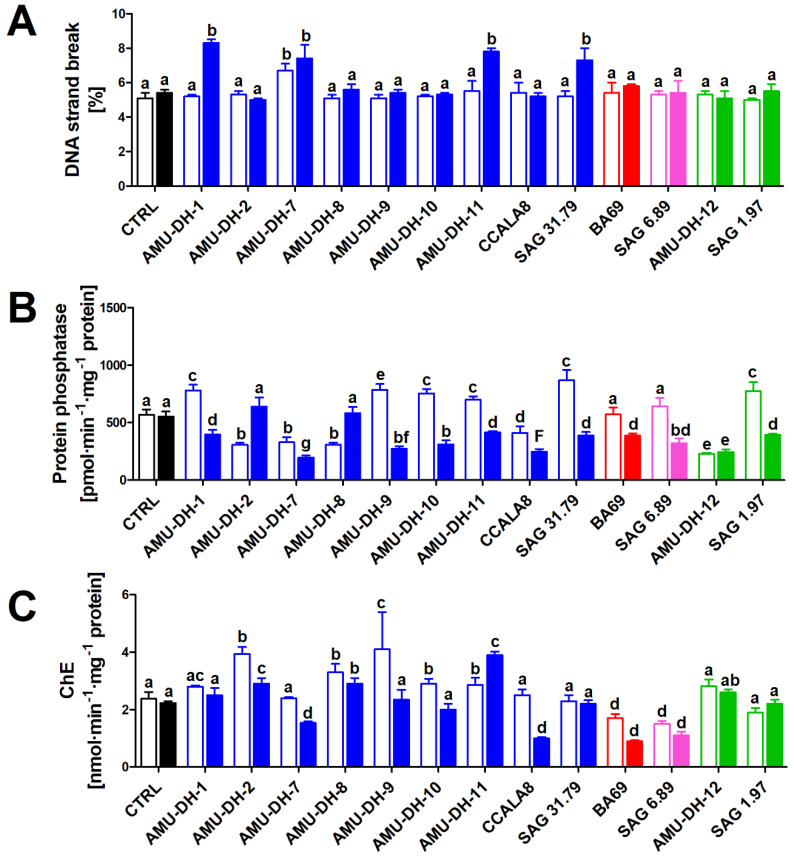
The effect (mean ± SD) of cyanobacterial extracts obtained from the 25- and 70-day-old cultures (open and filled bars, respectively) on DNA fragmentation (**A**) and serine/threonine protein phosphatase activity (**B**) in hepatocytes isolated from common carp, and cholinesterase activity in the brain homogenate (**C**) (*n* = 8). CTRL—control (cells/brain homogenate exposed to pure WC medium). Blue bars—*A. gracile* strains; red bars—*Aphanizomenon* sp. from the Baltic Sea; pink bars—*P. agardhii*; green bars—*R. raciborskii* strains. Different lowercase letters (a–g) above the bars indicate statistically significant difference between groups revealed by post hoc tests for pairwise comparisons (*p* < 0.05).

Most of the investigated extracts did not induce DNA strand breaks in carp hepatocytes. A significant increase in the rate of strand breaks was, however, noted following the exposure to extracts from the 25-day-old cultures of *A. gracile* AMU-DH-1, AMU-DH-7, AMU-DH-11, and SAG 31.79 (Figure 3).

### 2.3. Serine/Threonine Protein Phosphatase Activity

The studied extracts affected serine/threonine protein activity (Figure 3). In the case of *A*. *gracile* strains, an inhibition, in relation to control, was usually noted following the exposure to extracts obtained from the older cultures (AMU-DH-1, AMU-DH-7, AMU-DH-8, AMU-DH-10, AMU-DH-11, and SAG 31.79). A number of these strains (AMU-DH-1, AMU-DH-9, AMU-DH-10, AMU-DH-11, CCALA 8, and SAG 31.79) produced an opposite, stimulating effect on the 25th day of cultivation. The effects observed for the two tested strains of *R. raciborskii* revealed a distinctively different mode of action: AMU-DH-12 induced an inhibition during both culture developmental stages, while SAG 1.97 caused a stimulation on the 25th day of cultivation and decreased when the culture was 70 days old. Both *Aphanizomenon* sp. BA 69 and *P*. *agardhii* SAG 6.89 induced only an inhibition of protein activity—an effect noted exclusively for extracts originating from the 25-day-old cultures (Figure 3).

### 2.4. Neurotoxicity

The majority of the tested extracts induced changes in the cholinesterase activity in the fish brain homogenate. However, the magnitude and direction of these effects was highly dependent on the stage of culture development from which the extracts were obtained (Figure 3). Extracts of *A. gracile* strains AMU-DH-2, AMU-DH-8, and AMU-DH-11 induced an increase in the cholinesterase activity regardless of the stage of culture development, although for AMU-DH-2 and AMU-DH-8, the effect was more profound when the cultures were younger. The 25-day-old *A. gracile* AMU-DH-9 and AMU-DH-10 cultures induced an increase in the cholinesterase activity, while *A. gracile* AMU-DH-7 and CCALA 8 were capable of inhibiting it—this effect was, however, noted exclusively for extracts collected from the 70-day-old cultures. No effect was noted for *A. gracile* AMU-DH-1 and SAG 31.79 as well as *R. raciborskii* SAG 1.97, regardless of the stage of development. In the case of *Aphanizomenon* sp. BA 69 and *P. agardhii* SAG 6.89, a decrease in the cholinesterase activity was noted for extracts from both 25- and 70-day-old cultures, but the older cultures exerted a more profound effect (Figure 3).

## 3. Discussion

This study provides an insight into the toxicity profile during the growth of selected filamentous cyanobacteria. It is known that these microorganisms can produce numerous identifiable metabolites toxic to animals [19]. It was also established in laboratory cultures and in situ during blooms that the production of cyanotoxins often depends on the growth phase [37]. However, toxicological studies are usually focused on a single cyanotoxin, which allows exploring its exact mechanisms of action, whereas the massive occurrence of cyanobacteria in the aquatic environment is related to the presence of a mixture of various metabolites, some of which remain to be identified [33]. Therefore, the present study used extracts obtained from thirteen cyanobacteria cultures at two different developmental stages (25- and 70-day-old cultures). This, in turn, allowed revealing potential intra- and interspecific differences in the toxic potential during cyanobacterial growth.

Importantly, the extracts did not induce any effects in common carp cells in vitro that would reveal a generalized tendency regarding the stage of culture development at which they were the most toxic. In general, all examined extracts altered one or more of the considered parameters, indicating that they possess, to some degree, toxic potency. This highlights that the toxicity of cyanobacterial blooms may be challenging to predict based only on the development stage they are at. While studies monitoring intra- and extracellular levels of known cyanotoxins can provide an insight into the ecological and health risks, they cannot fully predict the toxicity which may originate from various compounds, including unknown metabolites. This finding underlines that cyanobacterial blooms should be considered as a threat regardless of their stage of development. 

The present study employed a number of assays to investigate different mechanisms of action possibly exhibited by the metabolites present in the extracts. It is known that a wide array of cyanobacterial compounds can induce an increased intracellular level of reactive oxygen species (ROS) in animal cells, eventually leading to a depletion of antioxidant capacity and detrimental cellular effects such as PC, lipid peroxidation, and damage to DNA [25,47,48,49,50]. The present study clearly demonstrated that redox balance in fish hepatocytes was disrupted following the treatment with the obtained extracts as indicated by the alterations in the GSH content, the activity of GST and CAT, and the level of TAC reflecting the overall balance between free radicals, antioxidant enzymes, and low molecular weight reducers. 

The effects exerted by a particular strain from the 25- and 70-day-old cultures varied widely depending on the studied parameter. Nevertheless, extracts of selected strains were capable of inducing oxidative damage. Lipid peroxidation, a chain reaction initiated by the hydrogen abstraction or the addition of oxygen radicals, predominantly generating genotoxic malondialdehyde and resulting in the oxidative damage of polyunsaturated fatty acids [51], was induced only in the 25-day-old *A. gracile* AMU-DH-11 and CCALA 8, and 70-day-old *R. raciborskii* AMU-DH-12. Extract of the younger *A. gracile* AMU-DH-11 also showed the greatest increase in protein carbonylation, a hallmark of oxidative damage [52]. The protein carbonylation was also elevated following the treatment with extract originating from the 70-day-old culture of this strain. The latter extract was capable of inducing DNA damage as well, as indicated by the increased rate of DNA strand breaks. Overall, it appears that the *A. gracile* AMU-DH-11 strain is of a particular concern with regards to its toxic potency. Genotoxic potency was also documented for *A. gracile* AMU-DH-7 regardless of the stage of culture development, as well as for the 70-day-old *A. gracile* strains AMU-DH-1 and SAG 31.79. Other cyanobacterial extracts did not induce a significant increase in DNA damage, although some revealed an effect on lipid peroxidation and protein carbonylation. Overall, these observations indicate that the studied cyanobacteria contain water-soluble metabolites, which can affect the redox balance and, in some cases, trigger the adverse outcomes of oxidative stress in fish hepatocytes. Importantly, strains of the same species—*A. gracile* and *R. raciborskii*—may differ from each other in terms of their exerted effects, indicating significant differences in levels of toxic compounds. Strain variability within a species was also postulated in earlier studies [53] as an explanation for the high variability of responses of isolates to the same environmental factors due to their varied physiological adaptation and toxic potency. These so-called ecotypes may vary in their adaptation across both large geographic regions and adjacent water bodies [54]. In addition, a high variation in toxicity has been shown to exist within strains forming blooms [55,56]. In light of these reports, the high strain variability observed in our study is unsurprising, and it clearly supports the importance of strain variation in understanding the influence of potentially toxic cyanobacteria on other organisms. An additional factor that might shape the toxicity of the examined strains is the bacterial activity in cyanobacterial cultures. Numerous studies have shown that bacterial communities accompany cyanobacterial blooms and can degrade cyanotoxins excreted by cyanobacteria into the external environment [57,58,59,60,61]. Their activity, however, may also be beneficial for cyanobacteria, and is partially responsible for the prolongation of cyanobacterial blooms and their strain variability [16]. 

Despite the observed high strain variability, it should be noted that the specific metabolites responsible for the observed effects are yet to be identified—none of the studied strains produced cylindrospermopsin (Table 1). Six out of the 13 strains were checked for MC-LR, MC-YR, and MC-RR, and these homologues of microcystins were detected only in *P. agardhii* SAG 6.89. Importantly, the toxicity of these hitherto unknown compounds to fish and invertebrates may vary. In the present study, extracts of the *A. gracile* strain SAG 31.79 and the *R. raciborskii* strain SAG 1.97 induced a toxic effect on fish cells, whereas previous research showed that exudates of these strains did not suppress life-history traits of *D. magna*, even if they were obtained from high-biomass cultures [62]. Moreover, it is worth noting that both *A. gracile* and *R*. *raciborskii* can be potent producers of saxitoxin alkaloids [63,64,65]. The present study did not investigate the production of these neurotoxic compounds. However, it has been previously reported that some strains of *A. gracile* and *R. raciborskii* are toxic despite a lack of synthesis of known compounds [25,66].

The present study also assessed the neurotoxic potential of cyanobacterial extracts, which was evaluated through the ChE activity in the brain homogenates. In general, the potential neurotoxic effects were more often observed for extracts originating from the 25-day-old cultures. An increased ChE activity was demonstrated for *A. gracile* AMU-DH-8, AMU-DH-2, AMU-DH-9, AMU-DH-10, AMU-DH-11, and *R. raciborskii* AMU-DH-12. This indicates that at the early stages of culture development, these strains produce compounds that can lead to a loss of cholinergic homeostasis and potentially result in rapid acetylcholine degradation and subsequent downregulation of acetylcholine receptors, leading to adverse effects on cognitive functions in fish [67]. Importantly, selected extracts from the older cultures of *A. gracile* AMU-DH-7 and CCALA 8, as well as *Aphanizomenon* sp. BA69 and *P. agardhii* SAG6.89, induced a significant inhibition of the ChE activity. Although it is known that *A. gracile* and *R*. *raciborskii* can produce neurotoxic Stx [63,64,65] and that their synthesis is higher in the younger cultures [68], these alkaloids are not known to affect the cholinesterase activity [69]. Its inhibition was, however, observed in response to anatoxin-a, a cyanotoxin produced by selected species belonging to Nostocales order, including *Aphanizomenon*, *Planktothrix*, and *Raphidiopsis* [70,71,72]. Whether the strains investigated in the present research are anatoxin-a producers would require further investigation. One should note that, as evidenced by the molluscan experimental model, Hungarian strains of *R. raciborskii* that do not produce anatoxin-a reveal anatoxin-like responses by modulating the acetylcholine receptors on neurons, evoking acetylcholine agonist effects, then inhibiting the acetylcholine-evoked neuronal responses [73,74]. Overall, the present study highlights the need to further explore the neurotoxicity of the investigated cyanobacterial species and strains.

## 4. Conclusions

In conclusion, the examined extracts of cyanobacteria from both 25- and 70-day-old cultures resulted in various alterations in *Cyprinus carpio* cells, including oxidative stress induction, DNA damage in hepatocytes, and changes in the activity of serine/threonine protein phosphatase in hepatocytes and cholinesterase in the brain homogenate. The stage of culture development from which the extracts were obtained was a significant factor influencing the toxicity to fish cells, although the effect varied from strain to strain. In the case of some studied strains, extracts from the 25-day-old cultures were more potent, while in the case of other strains, the opposite reactions or a lack of significant effect were observed. This suggests that there is no clear pattern regarding the degree of cyanobacterial bloom toxicity at a given stage of development. The present study also implies that some cyanobacterial blooms at the early, forming stages can already pose a toxic threat similar to those that are old with a tendency to collapse.

## 5. Materials and Methods 

### 5.1. Cyanobacterial Strains

Thirteen strains of filamentous cyanobacteria were cultivated for the purpose of this study: *A. gracile* (strains: AMU-DH-1, AMU-DH-2, AMU-DH-7, AMU-DH-8, AMU-DH-9, AMU-DH-10, AMU-DH-11, CCALA 8, SAG 31.79), *Aphanizomenon* sp. from the Baltic Sea (strain BA 69), *P. agardhii* (strain SAG 6.89), and *R. raciborskii* (strains: AMU-DH-12, SAG 1.97). Strain BA 69 was obtained from the Culture Collection of the Baltic Algae at the University of Gdańsk in Poland, strain CCALA 8 originated from Culture Collection of Autotrophic Organisms of the Institute of Botany of the Academy of Science of the Czech Republic, strains SAG 1.97, SAG 6.89, and SAG 31.79 were purchased from the Culture Collection of Algae at Göttingen University in Germany. The remaining strains were newly isolated from several lakes in Western Poland by L. Wejnerowski using the procedure described by Zapomělová and collaborators [75]. Strains were identified according to the morphological criteria provided by Komárek [76,77]. Table 1 summarizes the investigated strains and provides information on cyanotoxin production (if available), strain origin, and optical density of cultures measured at the wavelength of 750 nm using Epoch microplate spectrophotometer (BioTek Instruments, Winooski, VT, USA). All strains are currently maintained in a collection at the Department of Hydrobiology, Adam Mickiewicz University in Poznań, Poland.

Strains were monocyanobacterial, but not axenic, and cultured in WC medium [78] for 70 days since reinoculation in Erlenmeyer flasks. Cultures (300 mL) were maintained in a walk-in phytotron chamber (Conviron, Winniped, Canada) under defined light intensity (40–50 µmol photons m^–2^ s^–1^), photoperiod (16:8 hours light:dark cycle), and temperature (20 ± 0.5 °C). The 70-day-old cultures of *A. gracile* and *R. raciborskii* exhibited a symptom of culture aging visible to the eye, that is a slight change of culture color from greenish to olive-green or yellowish.

### 5.2. High Performance Liquid Chromatography Analyses

Toxin analyses of the cyanobacterial strains (AMU-DH-1, AMU-DH-2, CCALA 8, SAG 31.79, SAG 6.89, and SAG 1.97) were carried out by HPLC-DAD (diode array detection) available at UNESCO Chair on Ecohydrology and Applied Ecology at University of Łódź (Poland). Collected samples were filtered on a filtration system equipped with Whatman GF/C filters (0.47 mm diameter) at a volume from 65 to 100 mL, and cyanobacterial samples in the suspended material were extracted in 75% aqueous methanol for the MCs analyses and in 100% methanol for the CYN analyses. Samples were sonicated for 30 s in a Misonix (Farmingdale, NY, USA) ultrasonicator equipped with an ultrasonic probe (100 W, diameter 19 mm with a “spike”) and the liquid processor XL. The extracts were then centrifuged at 11,000× *g* for 10 min at 4 °C in the Eppendorf 5804 centrifuge (Hamburg, Germany). The supernatants were collected and evaporated to dryness in a SC110A Speedvac^®^ Plus, ThermoSavant (Holbrook, NY, USA). Before the HPLC analysis, the samples were redissolved in 500 μL of 75% aqueous methanol for the MCs analyses and in 500 μL of water for the CYN analyses, and filtered through a Gelman GHP Acrodisc 13 mm syringe filter with a 0.45 µm GHP membrane and a minispike outlet (East Hills, NY, USA). Chromatographic separation was performed using an Agilent (Waldbronn, Germany) 1100 series HPLC system consisting of a degasser, a quaternary pump, a column compartment thermostat set at 40 °C, and a diode array detector operated at 200–300 nm on a Merck (Darmstadt, Germany) Purospher STAR RP-18e column (55 × 4 mm I.D. with 5 μm particles) protected by a 4 × 4 mm guard column for the MCs analyses and a Purospher STAR RP-18e column (55 × 4 mm I.D. with 3 μm particles) protected by a 4 × 4 mm guard column for the CYN analyses. The mobile phase consisted of water (solvent A) and acetonitrile (solvent B), both containing 0.05% trifluoroacetic acid for the MCs and CYN analyses. The flow rate for the MCs analyses was 1 mL min^−1^ with the following linear gradient program: 0 min, 25% B; 5 min, 70% B; 6 min, 70% B; 6.10 min, 25% B. For the CYN analyses, the flow rate was 1.0 mL min^−1^ with the following linear gradient program: 0 min, 1% B; 5 min, 7% B; 5.1 min, 70% B; 7 min, 70% B; 7.1 min, 1% B. The injection volume was 20 μL in both cases. The three analogues of MCs (MC-LR, MC-YR, MC-RR) and CYN were identified by comparing the retention time and their characteristic absorption spectra with maximum at 238 nm for MCs, and 262 nm for CYN. 

All strains, with the exception of BA 69, were analyzed for CYN.SAG 6.89 was also analyzed for MCs by HPLC-DAD (Agilent 1100 series HPLC system) and HPLC-MS/MS (Agilent 1200 Rapid Resolution LC coupled to a Bruker Daltonics HCT ultra ion trap mass spectrometer with electrospray ion ESI source) available at Biochemistry, Faculty of Science and Engineering, Åbo Akademi University in Turku (Finland). We followed the protocol for CYN described in Kokociński et al. [79] and the protocol for MCs described in Hautala et al. [80].

### 5.3. Extract Preparation

The extracts were obtained from cultures grown for 25 and 70 days. Each extract was obtained from a separately grown culture. Fifty-milliliter samples were used for the preparation of cyanobacterial extracts. Culture volumes were sonicated at 4 °C using probe Ultrasonic Processor VCX 130 (Sonics, Newtown, CT., USA) until all cyanobacterial cells were disintegrated (verified under a light microscope). Homogenates were then filtered through 0.45 μm GF/C filters (Whatman, Maidstone, UK). Extracts were kept at −40 °C prior to the toxicological analyses. Simultaneously, cultures were subjected to chlorophyll-a concentration—20 mL of each culture was collected, filtered through GF/C filters (Whatman, Maidstone, UK), and analyzed spectrophotometrically following the extraction in 90% acetone [81]. The concentration of the extracts was standardized to be equal to 1400 μg chlorophyll per L^−1^ to allow a direct comparison of their toxic potencies.

### 5.4. Experimental Design

The toxicity of cyanobacterial extracts was tested in vitro in hepatocytes and the brain homogenate obtained from common carp *Cyprinus carpio*. The suitability of this experimental model in studies on cyanobacterial compounds and extracts was shown in previous studies [25,82].

The detailed protocol of hepatocytes and brain isolation was described in the publication of Evans and collaborators [82]. In brief, hepatocytes were isolated from common carp liver by digestion in 100 μL of 0.1% trypsin/EDTA for 2 min. The hepatocyte yield was around 56–82 × 10^6^ g^−1^ liver, and the cell viability was higher than 83%. In each experiment, resuspended cells (10^7^ cells per mL of Hank’s balanced salt solution) were exposed to 1% of each studied extract (related to chlorophyll content) for 2 h at 20 °C. The exposures to extracts obtained from two different ages of cyanobacterial culture were run simultaneously. The negative control was designed for cells unexposed to any cyanobacterial compounds. After the treatment, the isolated hepatocytes were washed in physiological saline, resuspended with trypsin, and kept for further analysis. The dissected brain tissues were homogenized at 4 °C in 0.1 M pH 7.4 phosphate buffer containing 100 mM KCl, 1 mM EDTA, and 0.1 mM PMSF (1:10 w:v) using an electric homogenizer with a Teflon pestle, and then centrifuged at 6000× *g* for 10 min at 4 °C. Selected parameters related to oxidative stress (total glutathione, the activity of glutathione-S-transferase and catalase, total antioxidant capacity, lipid peroxidation, protein carbonyl, catalase activity) and genotoxicity (DNA fragmentation) were tested in hepatocytes. Cholinesterase activity was tested in the brain homogenate. The analytical data were expressed by the protein content determined in whole cell lysates using the Lowry protein assay [83]. The control sample constituted of cells/brain homogenate exposed to the WC medium. Each assay was performed on eight independent cell cultures with each cell culture prepared from an individual fish.

#### 5.4.1. Oxidative Stress

Total glutathione (GSH) level in the isolated cells was estimated by the DTNB/glutathione reductase enzymatic recycling protocol [84]. The rate of 5-thionitrobenzoic acid formation was monitored spectrometrically at 405 nm. Standards were prepared from oxidized glutathione (GSSG), and the concentrations were expressed as nmol per mg of protein.

The activity of glutathione-S-transferase (GST, EC 2.5.1.18) in isolated hepatocytes was determined using the protocol by Habig et al. [85] based on the GST-catalyzed reaction between GSH and 1-chloro-2,4-dinitrobenzene (CDNB) as a substrate. The GST activity was monitored by registering an increase in the absorbance at 340 nm for 10 min and expressed as nmol per min per mg of protein.

Activity of catalase (CAT, EC 1.11.1.6) was evaluated in the supernatant of exposed hepatocytes by the decomposition of 10 mM H_2_O_2_ based on the protocol of Aebi and collaborators [86]. The decrease in the absorbance was registered at 240 nm and the CAT activity was calculated using the molar extinction coefficient (ε = 40 M^−1^ cm^−1^). 

Trolox equivalent antioxidant capacity (TEAC) was obtained with the ABTS assay, which is based on the inhibition of the absorbance of the 2,2′-azinobis (3-ethylbenzothiazoline 6-sulfonate; ABTS +) radical cation by cellular antioxidants [87]. The decrease in the absorbance was measured at 734 nm. A standard curve was obtained by using Trolox standard solution at the range of concentrations, from 0 to 0.24 μg/mL. The free radical scavenging capacity of the studied sample was presented as the percentage inhibition of ABTS in µM Trolox.

Lipid peroxidation (LPO) was determined from supernatants of isolated cells based on the reaction of malondialdehyde with 2-thiobarbituric acid. The absorbance of the pinkish colored complex of TBA-reactive substances (TBARS) was determined at 532 nm. The molar extinction coefficient of 1.56 × 10^5^ M^−1^ cm^−1^ was used. Results are presented as pmol TBARS mg^−1^ protein.

Protein carbonyl (PC) concentration was evaluated in the protein-rich pellet of the trichloroacetic acid-treated isolated cells by the reaction with 2,4-dinitrophenylhydrazine (DNPH) [88]. The PC level was calculated from the absorbance at 370 nm of the DNPH-derivatized samples in 8 M urea using the molar extinction coefficient of 2.2 × 10^4^ M^−1^ cm^−1^. Results were expressed as nmol PC mg^−1^ protein.

#### 5.4.2. DNA Fragmentation

DNA strand break was investigated in hepatocytes lysates exposed to the alkaline DNA precipitation assay using Hoechst 33342 [50]. The assay was performed in the presence of 4 mM sodium cholate to minimize the potential interference with traces of sodium dodecyl sulfate. The fluorescence was detected by an f-max (Ex/Em = 360/450 nm). Standard solutions of salmon sperm DNA were used for calibration. 

#### 5.4.3. Serine/Threonine Protein Phosphatase Activity 

Serine/threonine protein phosphatase activity was determined in isolated hepatocytes by colorimetric assay using p-nitrophenyl phosphate as a substrate [89]. The Protein Phosphatase activity was calculated from the absorbance at 405 nm using the molar extinction coefficient of 1.8 × 10^4^ M^−1^ cm^−1^. Results were expressed as pmol p-nitrophenol min^−1^ mg^−1^ protein.

#### 5.4.4. Cholinesterase Activity

Cholinesterase (ChE, EC 3.1.1.7) activity was determined in the exposed brain homogenate using the acetylthiocholine-cleaving ChE activity protocol based on the colorimetric method of Ellman et al. [90]. ChE was monitored by registering an increase in the absorbance at 405 nm for 10 min and calculated using the molar extinction coefficient of 13.6 × 10^3^ M^−1^ cm^−1^.

### 5.5. Statistical Analyses

Statistical analyses were performed using the Statistica v.12.0 (StatSoft, Tulsa, OK, USA). All data were normally distributed and met the assumption of the equality of variances (tested with Kolmogorov-Smirnov and Levene tests, respectively). The difference between effects of separate extracts on the studied biological targets were tested with one-way ANOVA with the Tukey HSD post hoc test (α = 0.05). Two-way ANOVA was applied to assess the contribution of each of the factors, namely the stage of development and the studied cyanobacterial strains, as well as to determine the significance of their interaction.

## Figures and Tables

**Table 1:** Data on cylindrospermopsin (CYN) and three homologues of microcystin (MC-RR, MC-YR, MC-LR) in the investigated strains of cyanobacteria, strain origin, optical density (mean ± SD), and chlorophyll concentration in cultures. Symbols + and – indicate a detected and nondetected toxin, respectively. *n.e*. indicates that we do not have data on the presence/absence of a given toxin. The lack of data on the toxicity of some strains was due to technical reasons (strains have been isolated/obtained later than HPLC toxin analyses have been performed).

Species, Strain	CYN	MC-RR	MC-YR	MC-LR	Source of Information	Origin	Optical Density (750 nm) in Cultures (Chlorophyll Concentration in µg L^−1^)		
							Day 0	Day 25	Day 70
*A. gracile*									
AMU-DH-1	–	–	–	–	this study	Lake Buszewskie (52°32′42″ N, 16°22′47″ E)	0.008 ± 0.001	0.819 ± 0.095 (2376)	0.774 ± 0.136 (3729)
AMU-DH-2	–	–	–	–	[46]	Lake Lubosińskie (52°31′40″N, 16°22′56″E)	0.006 ± 0.001	0.712 ± 0.078 (3814)	0.682 ± 0.232 (4599)
AMU-DH-7	–	*n.e.*	*n.e.*	*n.e.*	this study	Lake Pniewskie (52°30′41″N, 16°14′27″)	0.057 ± 0.006	0.654 ± 0.12 (2477)	0.689 ± 0.294 (5731)
AMU-DH-8	–	*n.e.*	*n.e.*	*n.e.*	this study	Lake Pniewskie (52°30′41″N, 16°14′27″)	0.008 ± 0.002	0.619 ± 0.057 (1733)	0.755 ± 0.129 (2161)
AMU-DH-9	*–*	*n.e.*	*n.e.*	*n.e.*	this study	Lake Pniewskie (52°30′41″N, 16°14′27″)	0.06 ± 0.024	0.604 ± 0.066 (2329)	0.607 ± 0.107 (4164)
AMU-DH-10	–	*n.e.*	*n.e.*	*n.e.*	this study	Lake Pniewskie (52°30′41″N, 16°14′27″)	0.008 ± 0.001	0.491 ± 0.042 (5100)	0.594 ± 0.243 (4362)
AMU-DH-11	–	*n.e.*	*n.e.*	*n.e.*	this study	Lake Pniewskie (52°30′41″N, 16°14′27″)	0.007 ± 0.005	0.448 ± 0.122 (2508)	0.653 ± 0.161 (2385)
CCALA 8	–	–	–	–	[46]	Lake Lough Neagh (54°37’06’’N, 6°23’43’’E)	0.008 ± 0.002	0.666 ± 0.121 (1415)	0.762 ± 0.131 (3272)
SAG 31.79	–	–	–	–	[46]	Lake Plußee (54°11’00.7’’N, 10°26’45.9’’E)	0.008 ± 0.001	0.726 ± 0.147 (2719)	0.679 ± 0.189 (3794)
*Aphanizomenon* sp.									
BA 69	*n.e.*	*n.e.*	*n.e.*	*n.e.*		Puck Bay, Baltic Sea	0.008 ± 0.001	0.553 ± 0.272 (2545)	0.809 ± 0.248 (3505)
*P. agardhii*									
SAG 6.89	–	+	+	+	[46]	Lake Plußee (54°11’00.7’’N, 10°26’45.9’’E)	0.007 ± 0.002	0.46 ± 0.067 (1416)	0.61 ± 0.146 (2625)
*R. raciborskii*									
AMU-DH-12	–	*n.e.*	*n.e.*	*n.e.*	this study	Lake Pniewskie (52°30′41″N, 16°14′27″)	0.008 ± 0.001	0.586 ± 0.09 (2851)	0.582 ± 0.199 (2186)
SAG 1.97	–	–	–	–	[46]	Lake Balaton (46°48’51.0’’N, 17°45’52.8’’E)	0.013 ± 0.003	0.459 ± 0.064 (2705)	0.819 ± 0.171 (3151)

**Table 2:** Results of two-way ANOVA for the effect of the stage of culture development, strain, and their interaction on the glutathione level (GSH), glutathione S-transferase activity (GST), catalase activity (CAT), total antioxidant capacity (TAC), lipid peroxidation (TBARS), protein carbonylation (PC), DNA strand breaks (DSBs), serine/threonine protein phosphatase activity (PSP) in hepatocytes, and cholinesterase activity (ChE) in the brain homogenate of *C. carpio*.

Studied Biomarker	Stage of Culture Development	Strain	Stage of Culture Development × Strain
*GSH*	F_1,140_ = 250.3; *p* < 0.001	F_13,140_ = 13.1; *p* < 0.001	F_13,140_ = 10.0; *p* < 0.001
*GST*	F_1,140_ = 148.3; *p* < 0.001	F_13,140_ = 8.7; *p* < 0.001	F_13,140_ = 10.2; *p* < 0.001
*CAT*	F_1,140_ = 36.6; *p* < 0.001	F_13,140_ = 3.6; *p* < 0.001	F_13,140_ = 5.5; *p* < 0.001
*TAC*	F_1,140_ = 0.3; *p* = 0.601	F_13,140_ = 11.6; *p* < 0.001	F_13,140_ = 20.6; *p* < 0.001
*TBARS*	F_1,140_ = 35.3; *p* < 0.001	F_13,140_ = 3.7; *p* < 0.001	F_13,140_ = 2.6; *p* < 0.01
*PC*	F_1,140_ = 385.3; *p* < 0.001	F_13,140_ = 33.8; *p* < 0.001	F_13,140_ = 25.1; *p* < 0.001
*DSBs*	F_1,140_ = 24.6; *p* < 0.001	F_13,140_ = 6.8; *p* < 0.001	F_13,140_ = 3.9; *p* < 0.001
*PSP*	F_1,140_ = 124.9; *p* < 0.001	F_13,140_ = 14.7; *p* < 0.001	F_13,140_ = 12.4; *p* < 0.001
*ChE*	F_1,140_ = 38.6; *p* < 0.001	F_13,140_ = 26.6; *p* < 0.001	F_13,140_ = 6.8; *p* < 0.001
